# Case Studies and Literature Review of Pneumococcal Septic Arthritis in Adults

**DOI:** 10.3201/eid2510.181695

**Published:** 2019-10

**Authors:** Amandine Dernoncourt, Youssef El Samad, Jean Schmidt, Jean Philippe Emond, Charlotte Gouraud, Anne Brocard, Mohamed El Hamri, Claire Plassart, Florence Rousseau, Valéry Salle, Momar Diouf, Emmanuelle Varon, Farida Hamdad

**Affiliations:** Amiens-Picardie University Medical Center, Amiens, France (A. Dernoncourt, Y. El Samad, J. Schmidt, J.P. Emond, A. Brocard, M. El Hamri, C. Plassart, F. Rousseau, V. Salle, M. Diouf, F. Hamdad);; Compiègne Hospital, Compiègne, France (J.P. Emond, C. Gouraud);; Senlis Hospital, Senlis, France (A. Brocard);; Laon Hospital, Laon, France (M. El Hamri); Beauvais Hospital, Beauvais, France (C. Plassart);; Intercommunal Hospital of Creteil, Paris, France (E. Varon)

**Keywords:** *Streptococcus pneumoniae*, septic arthritis, pneumococcal disease, vaccines, bacteria, France

## Abstract

We conducted a retrospective study on all cases of pneumococcal septic arthritis (SA) in patients >18 years of age reported to the Picardie Regional Pneumococcal Network in France during 2005–2016. Among 1,062 cases of invasive pneumococcal disease, we observed 16 (1.5%) SA cases. Although SA is uncommon in adult patients, the prevalence of pneumococcal SA in the Picardie region increased from 0.69% during 2005–2010 to 2.47% during 2011–2016 after introduction of the pneumococcal 13-valent conjugate vaccine. We highlight the emergence of SA cases caused by the 23B serotype, which is not covered in the vaccine.

## Abstract 2

We saw an increase in this condition related to emergence of *Streptococcus pneumoniae* serotype 23B.

Septic arthritis (SA) constitutes a medical emergency and is associated with high rates of illness and death (*1*–*3*). The annual incidence of proven or probable SA in industrialized countries is 4–10/100,000 patients in the general population and 30–70/100,000 in patients with rheumatoid arthritis or history of prosthetic joint replacement surgery (*1*–*3*). The increasing prevalence over recent decades is related to an aging population, use of immunosuppressive drugs, and the growing number of orthopedic procedures (*1*–*3*). Large studies of SA have identified *Staphylococcus aureus* as the most common is organism involved, along with *Streptococcus pyogenes* to a lesser degree (*1*–*4*). *Streptococcus pneumoniae* is considered an uncommon cause of SA in adults (*3*–*11*).

*S. pneumoniae* is a common cause of bacterial community-acquired pneumonia, acute otitis, maxillary sinusitis, and severe invasive infections, especially in patients <2 or >65 years of age and in patients with underlying conditions, such as diabetes, malignancy, immune deficiency, chronic alcoholism, and splenectomy (*12*–*15*). Invasive pneumococcal disease (IPD) is a major public health concern worldwide, with a reported incidence of 7–97/100,000 persons >18 years of age annually (*14*). IPD is defined as isolation of pneumococci from normally sterile body fluids. According to a review from 1952–2008, pneumococcal SA occurred in 0.6%–2.2% of all cases of IPD (*14*). Similarly, in a large case study published by Marrie et al. (*16*), 1.6% of patients with IPD had pneumococcal SA.

Antimicrobial drug therapy and vaccination have been central elements of the clinical approach to pneumococcal disease. Literature from the 1990s also emphasized the spread of pneumococcal strains resistant to β-lactams and other antimicrobial agents (*1*,*2*,*5*–*10*,*12*–*15*). Pneumococcal vaccination has since become a global public health focus (*15*,*17*–*21*).

We analyzed all cases of pneumococcal SA in patients >18 years of age reported to the Picardie Regional Pneumococcal Network in France during 2005–2016. We also reviewed scientific publications on SA from the 1950s through 2017. Our aim was to the determine prevalence of *S. pneumoniae* in SA and assess whether introduction of pneumococcal 13-valent conjugate vaccine (PCV13) might contribute to increased rates of pneumococcal SA.

## Patients and Methods

The Picardie region of France has a population of ≈2 million. Cases of IPD are reported to the Picardie Regional Pneumococcal Network, a collection of 12 public hospital-based clinical bacteriology departments across the region. We conducted a retrospective study of pneumococcal SA in patients >18 years of age reported to the network during January 1, 2005–December 31, 2016. We defined cases by either an *S. pneumoniae*–positive culture from synovial fluid, an *S. pneumoniae*–positive blood culture with purulent or inflammatory joint fluid, medical imaging consistent with the diagnosis of arthritis, or a combination of these.

We collected patient demographic characteristics, including age, sex, and underlying conditions; clinical signs and symptoms; whether patients had other sites of pneumococcal infection; laboratory findings; antimicrobial therapy; and clinical outcomes. We performed statistical analyses by using SAS version 9.4 (SAS Institute Inc., https://www.sas.com) software, and estimated p values for comparison of relative frequencies by using χ^2^ test. We considered p<0.05 the threshold for statistical significance. This study was conducted in compliance with French legislation and the Declaration of Helsinki regarding ethics principles for medical research involving human subjects.

We also conducted a review of the largest case studies (>3 patients) of pneumococcal SA in patients >18 years of age published in the medical literature during 1950–2017 by using the search terms “*Streptococcus pneumoniae* infection” and “Septic arthritis” in the PubMed database. We excluded studies reporting on patients <18 years of age, in vitro and animal studies, and other factors. We retrieved 15 studies for full-text review and identified 7 studies meeting our inclusion criteria (Figure).

**Figure:**
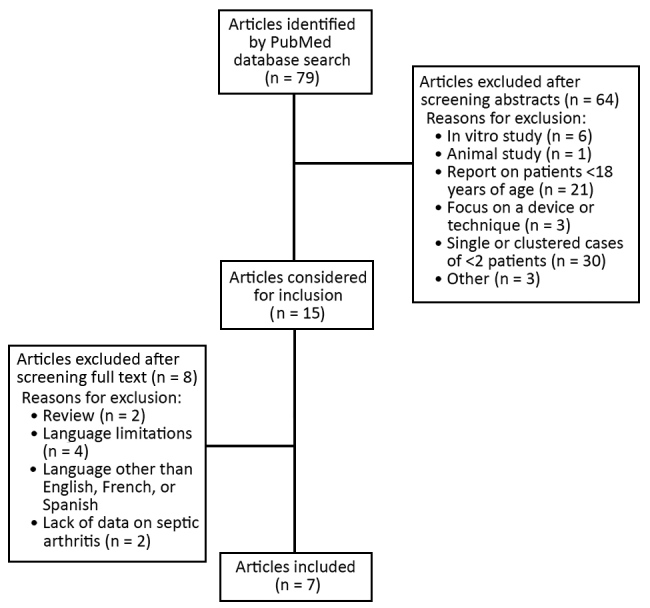
Selection process for systematic review of published data on pneumococcal septic arthritis in adults.

## Results

During January 1, 2005–December 31, 2016, we observed 16 (1.5%) cases of pneumococcal SA out of 1,062 cases of IPD reported (Table 1). The prevalence of pneumococcal SA increased during the study period, ranging from 0.69% during 2005–2010 to 2.47% during 2011–2016 (p = 0.02).

**Table 1:** Cases of septic arthritis and IPD, Picardie region, France, 2005–2016*

Year	2005	2006	2007	2008	2009	2010	2011	2012	2013	2014	2015	2016
IPD cases, no.	130	39	108	92	141	67	83	58	106	60	89	89
No. (%) pneumococcal SA	0	0	3 (2.8)	0	0	1 (1.5)	1 (1.2)	3 (5.2)	1 (0.9)	2 (3.3%)	0	5 (5.6)

*IPD, invasive pneumococcal disease; SA, septic arthritis

Of patients with pneumococcal SA, the mean age was 69.7 (34–93) years, and 62.5% were >65 years of age; 9 were women and 7 were men. Fourteen (87.5%) patients had monoarticular infection; the other 2 (12.5%) cases had polyarticular infection, 1 case involving the knee and shoulders and the other sacroiliac joints. 

For 14 patients, data on medical history, clinical characteristics, and time to diagnosis were available (Table 2). We found that 11 (78.57%) patients had 1–2 underlying conditions predisposing them to pneumococcal SA, 5 (35.71%) had 1 condition, and 6 (42.86%) had >1 condition; 3 (21.42%) had no risk factors but were among the oldest patients, and 5 (35.71%) had concurrent respiratory tract infections. Most joint infections involved the knee, 11/16 (68.75%) SA cases; 9 (56.25%) patients had native joint infections; and 5 (31.25%) patients had infections after prosthetic joint surgery, 4 involving a knee and 1 involving a hip. 

**Table 2:** Demographic data, clinical characteristics, underlying conditions, and other sites of pneumococcal infection for 16 patients with pneumococcal septic arthritis in the Picardie region, France, 2005–2016*

Patient age, y/sex	Affected joint	Days from admission to diagnosis	Underlying conditions, risk factors	Vaccination, type	Clinical signs and symptoms	Other infection
90/M	Hip, prosthetic joint surgery 1 y before SA	60	Multiple myeloma diagnosed 2 y after septic arthritis	ND	Joint pain	None
93/F	Knee, prosthetic joint surgery 5 y before SA	2	None	ND	Joint pain and swelling	Pneumonia
34/M	Hip	1	Chronic alcoholism	N	Fever, joint pain	None
68/M	Knee	4	Gout, heart disease	N	Fever, joint pain and swelling	Hematoma in abdominal muscles
75/M	Knee	1	Diabetes mellitus, heart disease	N	Joint pain and swelling	None
42/F	One knee, both shoulders	1	Splenectomy, Immunosuppressive drug	Y, PPV23	Joint pain and swelling	None
80/F	Knee	1	Chronic alcoholism	N	Joint pain and swelling	None
61/F	Knee	2	COPD, multiple myeloma diagnosed while hospitalized for septic arthritis	N	Fever, joint pain and swelling	None
57/F	Shoulder	1	COPD, MGUS	N	Joint pain and swelling	Bronchitis 3 weeks prior
82/F	Knee, prosthetic joint	ND	ND	ND	ND	ND
90/F	Knee	ND	ND	ND	ND	ND
53/F	Both sacroiliac joints	2	Splenectomy	Y, PCV13	Fever, joint pain	Sinusitis 3 weeks prior
69/M	Knee, prosthetic joint surgery 1 mo before SA	1	Malignant disease MGUS	N	Fever, joint pain and swelling	None
82/F	Knee, prosthetic joint surgery 1 y before SA	1	None	N	Fever, joint pain and swelling	Pneumonia
57/M	Knee	1	Chronic alcoholism	ND	Fever, joint pain and swelling	None
82/M	Sacroiliac joint	5	None	N	Joint pain	Pneumonia

*COPD, chronic obstructive pulmonary disease; MGUS, monoclonal gammopathy of undetermined significance; SA, septic arthritis; ND, no data.

Of note, 1 patient had undergone surgery for inguinal hernia, which was complicated by abdominal wall hematoma and pneumococcal SA with concomitant crystal-induced arthritis (gout). Another patient received a diagnosis of multiple myeloma while hospitalized for pneumococcal SA. An additional patient developed pneumococcal SA 2 years after the joint infection. 

Vaccination status was available for 11 (78.57%) of the 14 patients with clinical data. Only 2 patients had been vaccinated against pneumococci, both after splenectomy. 

Two patients were admitted to the intensive care unit with septic shock and severe renal failure. All patients reported pain of the infected joint; only half were febrile (>38°C) at admission; and 10 (71.42%) patients had joint swelling. The median interval between admission and diagnosis was 1 day (range 1–60 days). The patient with infection of the prosthetic hip joint had few symptoms, which might explain the delay in diagnosis of pneumococcal SA, which took 60 days.

Leukocyte scintigraphy was helpful for 3 patients, 1 with prosthetic hip joint infection and 2 with sacroiliac joint infection. Positron emission tomography was performed for a patient with infection of a single sacroiliac joint, which showed increased radionuclide uptake.

We noted increased white cell count, >10,000/mm^3^, in 13 (81.25%) cases with a mean of 13,800 cells/mm^3^ (range 6,020–78,000 cells/mm^3^). Fourteen patients had serum C-reactive protein (CRP) results, and 13 (92.82%) had CRP >100 mg/L (mean 325 mg/L [range 28–552 mg/L]) (Table 3). Procalcitonin test (PCT) was performed on 8 patients, and 6 (75%) had levels >0.5 ng/mL. Urinary pneumococcal antigen detection was performed for 5 patients, and 4 (80%) patients had positive results. 

**Table 3:** Laboratory findings, joint analysis, antimicrobial susceptibility, and serotypes of Pneumococcus-positive cultures for 16 patients with pneumococcal septic arthritis, Picardie region, France, 2005–2016*

Patient age, y/sex	Laboratory findings						Joint analysis				MIC, mg/L			Serotype
	CRP†	Leuk‡	PCT§	Bact	Ag U		Characteristics	Gram stain	Culture		PEN	AMOX	CEF	
90/M	100	10,400	0.14	Y	+		Inflammation	–	+		2	2	0.5	19F
93/F	505	11,900	5.6	Y	+		Purulent	+	+		0.016	0.016	0.016	3
34/M	290	14,000	0.36	N	NA		Purulent	+	+		0.016	0.016	0.016	10A
68/M	28	78,000	NA	Y	NA		Purulent	+	+		0.5	0.25	0.25	1
75/M	360	13,800	NA	Y	NA		Purulent	+	+		0.064	0.016	0.016	NA
42/F	203	44,000	13	Y	NA		Inflammation	–	+		0.016	0.016	0.016	23B¶
80/F	385	18,200	NA	Y	NA		Purulent	+	+		0.016	0.016	0.032	23B¶
61/F	552	6,020	155	Y	NA		Purulent	+	+		0.5	0.5	1	1
57/F	391	11,310	NA	N	NA		Inflammation	NA	+		0.008	0.016	0.016	23B¶
82/F	NA	12,200	NA	N	NA		Inflammation	+	+		0.016	0.016	0.032	6A#
90/F	NA	NA	NA	Y	+		Purulent	+	–		1	0.5	4	19F
53/F	110	12,800	69.9	Y	–		NA	NA	NA		0.016	0.016	0.016	24F¶
69/M	552	8,740	NA	Y	NA		Purulent	+	+		0.016	0.016	0.016	9N
82/F	178	19,480	3.72	Y	NA		Purulent	+	+		0.016	0.016	0.016	8
57/M	450	15,550	NA	N	NA		Purulent	–	+		0.032	0.016	0.016	23B¶
82/M	240	21,000	0.96	N	+		Inflammation	+	+		0.016	0.016	0.016	23B¶

*Ag U, pneumococcal antigen in urine; AMOX, amoxicillin; Bact, bacteremia; CEF, ceftriaxone; CRP, C-protein reactive; leuk, leukyocytes; NA, not available; PCT, procalcitonin; PEN, penicillin; PCV13, pneumococcal 13-valent conjugate vaccine; PPV23, pneumococcal 23-valent polysaccharide vaccine; +, positive; –, negative. †In mg/L. ‡In cells/mm^3^. §In ng/mL. ¶Serotypes not covered by PCV13 or PPV23.  #Serotype covered by PCV13 but not by PPV23.

Joint aspiration was performed in 15 cases; a patient with sacroiliac joint SA was excluded. All joint aspirates had white blood cell counts >10,000/mm^3^ on cytological analysis and were purulent in 10 (66.67%) cases. Gram staining showed gram-positive cocci in 11 (73.33%) cases. Joint fluid was cultured for *S. pneumoniae*, and 14 (93.33%) cases had positive cultures; *S. pneumoniae* strains were recovered from both joint aspirate and blood cultures from 9 (56.25%) cases. In 2 (12.5%) cases, bacteriological diagnosis of arthritis was made exclusively on the basis of blood culture, and in 5 (31.25%) cases, the positive culture was only obtained for joint aspirate.

Of the 16 *S. pneumoniae* strains we recovered, 4 (25%) had low-level penicillin resistance, 1 (6.25%) also had low-level ceftriaxone resistance, and 1 (6.25%) had high-level ceftriaxone resistance (MIC 4 mg/L). Three (75%) of 4 strains from 2005–2010 had low-level penicillin resistance, whereas only 1 (8.3%) of 12 strains from 2011–2016 had low-level penicillin resistance (p<0.01). All strains isolated from cases of nonbacteremic SA were penicillin susceptible. We serotyped 15 isolates and found 33.33% were strain 23B, 13.33% were 19F, and 13.33% were serotype 1 (Table 3). Serotype 23B was always penicillin susceptible, but other the serotypes had low-level penicillin resistance.

All patients were treated with a combination of 2 intravenous antimicrobial drugs, mainly amoxicillin and gentamicin (68.75%). The median duration for intravenous antimicrobial therapy was 6 days (range 1–27 days). After intravenous antimicrobial drug therapy, patients were prescribed oral antimicrobial drugs, such as amoxicillin, rifampin, levofloxacin, or clindamycin, alone or in combination (Table 4). The median overall duration of antimicrobial therapy was 42 days (range 42–84 days) for patients with native joint infection and 47 days (range 42–120 days) for patients with infection in prosthetic joints. In addition to antimicrobial therapy, all patients with prosthetic joint infection also underwent surgical drainage, and 1 patient also required replacement of the prosthetic hip joint.

**Table 4:** Clinical data on 16 patients with pneumococcal septic arthritis, Picardie region, France, 2005–2016*

Patient age, y/sex	Antimicrobial drugs, initial intravenous therapy; duration, d	Antimicrobial drugs, change to oral therapy	Surgical intervention	Duration of antimicrobial therapy, d	Clinical outcome
90/M	Ceftriaxone and rifampin; 4	Levofloxacin and rifampin	Arthrotomy and replacement of prosthetic joint	42	Recovered, regained baseline joint function
93/F	Ceftriaxone and gentamicin; 5	Levofloxacin and clindamycin	Arthrotomy and synovectomy	120	Recovered, regained baseline joint function
34/M	Amoxicillin and gentamicin; 4	Levofloxacin and amoxicillin	None	42	Recovered, moderately reduced range of motion
68/M	Amoxicillin and gentamicin; 2	NA	None	2	Died 2 d after admission from multiorgan failure related to colchicine overdose
75/M	Amoxicillin and gentamicin, then amoxicillin and levofloxacin; 11	Levofloxacin	None	42	Recovered, regained baseline joint function
42/F	Vancomycin and gentamicin; 6	Levofloxacin and rifampin	Arthrotomy	42	Recovered, moderately reduced range of motion
80/F	Ofloxacin and cloxacillin; 1	Amoxicillin	None	42	Recovered, regained baseline joint function
61/F	Amoxicillin and levofloxacin and rifampin; 7	Amoxicillin and rifampin	Arthrotomy	42	Recovered, regained baseline joint function
57/F	Amoxicillin and gentamicin; 5	Amoxicillin	None	42	Recovered, moderately reduced range of motion
82/F	ND	ND	ND	ND	ND
92/F	ND	ND	ND	ND	ND
53/F	Ceftriaxone and gentamicin; 15	Levofloxacin	None	84	Recovered, regained baseline joint function
69/M	Amoxicillin and gentamicin; ND	ND	ND	ND	Recovered, regained baseline joint function
82/F	Cefotaxime and gentamicin; 7	Levofloxacin and rifampin	Arthrotomy	47	Recovered, moderately reduced range of motion
57/M	Amoxicillin and gentamicin; 27	Amoxicillin	Arthrotomy and synovectomy	42	Recovered, moderately reduced range of motion
82/M	Amoxicillin and gentamicin; 10	Amoxicillin	None	42	Recovered, regained baseline joint function

*NA, not applicable; ND, no data.

Most patients survived, but 1 (6.25%) patient died from colchicine-related multiorgan failure 2 days after admission. The remaining patients recovered well 8 (57.14%) of 14 patients completely recovered, and 5 (35.71%) had moderately reduced range of motion in the affected joint (Table 4).

## Literature Review

We reviewed the largest case studies published during 1950–2017 (Table 5) and identified 121 cases of *S. pneumoniae* SA in adults in the literature (*4*,*6*–*10*,*16*). The age of affected patients was 47–75 years. Case-patients included 71 men and 50 women, 87.6% (106/121) of whom had underlying conditions that might have been predisposing factors for pneumococcal SA, including rheumatoid arthritis, gout and degenerative joint disease, diabetes, alcoholism, immunosuppression, cardiovascular disease, chronic obstructive pulmonary disease, malignancy, corticosteroid use, and splenectomy.

**Table 5:** Detailed analysis of reports of septic arthritis in patients >18 years of age reported in medical literature during 1950–2019*

Study details	Study authors, year published (reference)							
	This study	Ros, et al., 2003 (*10*)	Ispahani et al., 1999 (*8*)	Dubost et al., 2004 (*4*)	Raad et al., 2004 (*6*)	James et al., 2000 (*7*)	Belkhir et al., 2014 (*9*)	Marrie et al., 2017 (*16*)
No. patients	16	11	25	7	4	14	9	51
Median age, y (IQR)	69.7 (+25)	60 (+18.5)	69 (NA)	63 (NA)	47 (+3.25)	63 (+21.75)	75 (+26)	56 (+17)
Sex, M/F	7/9	6/5	11/14	4/3	2/2	9/5	4/5	35/16
Impaired joints, no.								
Knee	11	6	12	4	0	10	7	26
Hip	2	0	0	0	2	0	0	0
>1 joint	2	3	5	0	3	6	0	9
Prosthetic joint	5	2	2	0	0	0	0	NA
Extraarticular infections, no.	6†	5	11	1	2	7	5	NA
Underlying conditions, no.	11†	9	23	5	4	10	3	48
Known vaccination status, no.	11	0	0	0	0	0	1	0
No. immunized	2	–	–	–	–	–	0	–
Laboratory data, no. positive/no. tested								
Bacteremia	11/16	9/11	20/24	4/6	1/4	8/14	5/9	51/51
Gram stain	11/14	NA	23/25	4/5	NA	NA	5/9	NA
Culture	14/15	11/11	19/25	7/7	4/4	13/14	7/9	NA
Serotypes, no.	6	1	1	NA	NA	NA	4	29
Strains	23B and 24F	6A	24F	–	–	–	NA	NA
Covered by PCV13 and PPV23	N	Y	N	–	–	–	Y	
Susceptibility								
Penicillin S	4	3	25	NA	2	NA	5	NA
Ceftriaxone S	1	1	25	NA	1	1	NA	NA
Ceftriaxone R	1	–	–	–	–	–	NA	NA
Median duration of antimicrobial therapy, d	42	42	49	27	49	NA	44.7	NA
Surgical intervention, no.‡	6†	8†	21	3	2	11	6	NA
Clinical outcome								
Death	1	2	8	0	0	3	1	6
Sequelae	5	4	10	4	1	2	1	NA

*IQR, interquartile range; NA, not available; PCV13, pneumococcal 13-valent conjugate vaccine; PPV23, pneumococcal 23-valent polysaccharide vaccine; R, resistant; S, susceptible. †Data not available for all patients in study. ‡Including arthrotomy or synovectomy of affected joint or prosthetic joint replacement.

The clinical characteristics of pneumococcal infection, laboratory findings, antimicrobial therapy, and clinical outcomes were not available for all cases, and immunization status rarely was described. Among 70 patients for whom clinical characteristics were available, 36 (51.42%) had either prior or concurrent pneumococcal infections other than SA, including 24 (35.71%) cases of pneumonia, 10 (14.29%) cases of meningitis, and 4 (5.71%) cases of endocarditis. The knee was the joint most commonly involved in SA (66/121), but other affected sites included the shoulder (19/105), ankle (11/105), hip (10/112), and elbow (9/105). Polyarticular involvement was reported in 23% of patients (28/121). Of 70 patients with prosthetic joint replacement, 4 (5.71%) had *S. pneumoniae* infections and 61 (87.14%) had joint cultures that were positive for bacteremia. 

Concomitant bacteremia was documented in 98/119 (82.35%) patients for whom blood culture results were reported. Of 59 documented isolates, 5 (8.47%) demonstrated low-level penicillin resistance and 3 (5.1%) had low-level ceftriaxone resistance. Serotype data were seldom available, but a study by Marrie et al. (*16*) listed serotypes 4, 8, and 22F as the most commonly isolated. 

Of the 121 patients we identified, 117 (96.7%) received antimicrobial therapy. Of those, 41 cases had detailed data on treatment regimens. Penicillin was the first-line treatment in 29 (70.7%) cases; third-generation cephalosporins, vancomycin, and rifampin were administered less frequently. 

Among the 63 patients for whom clinical outcome data were available, 58 (92.1%) underwent joint drainage, and 11.1%–66.7% experienced sequelae of the joint infection. Death rates were variable among the studies but ranged as high as 32%.

## Discussion

We report 16 (1.5%) cases of pneumococcal SA in a cohort of 1,062 IPD patients in France. Our study only includes data on *S. pneumoniae*–positive cultures from patients who were treated in public-sector hospitals. The true number of cases of pneumococcal SA in the region likely would be higher if data from private-sector hospitals were included. We found that the prevalence of pneumococcal SA reported to the Picardie Regional Pneumococcal Network increased 4-fold after introduction of PCV13 in 2010.

We observed slight female predominance in our case-patients, which is in line with other studies (*8*,*9*). However, some studies describe male predominance (*4*,*7*,*10*,*16*). We found that older adults were more susceptible to pneumococcal SA; 62.5% of our patients were >65, but this proportion was lower than reported in previous studies (*4*,*6*–*10*,*16*,*22*). Underlying conditions that could predispose patients to pneumococcal SA were observed in 79% of cases, and 21% of pneumococcal joint infections occurred in apparently healthy patients, which is in line with the results reported by others (*23*).

Among our cohort, 2 cases of multiple myeloma were revealed by pneumococcal SA. Because multiple myeloma causes immunosuppression, patients with multiple myeloma are more likely to become infected by encapsulated bacteria. Clinicians should consider multiple myeloma when pneumococcal SA is diagnosed in patients with no apparent predisposing factor (*24*,*25*). 

Some studies report a history of concomitant extra-articular infection in 40%–60% of patients with pneumococcal joint infection (*6*–*8*,*10*). We noted pneumococcal respiratory tract infection in 36% of our cohort, but the respiratory tract was the only site of concomitant extra-articular infection reported. The knee was the joint affected most commonly. Among our cohort, 62.5% had SA in the knee and 12.5% had SA in the hip, a site reported less commonly overall; only 1%–7.8% of SA cases in the literature involved the hip (*9*,*10*,*26*). SA involved a prosthetic joint in 31% of cases, a higher rate than previously reported (*6*,*7*,*9*,*10*,*26*). Joint prosthesis in older adults appears to be an added risk factor for SA.

For most patients, the time to diagnosis was a few days. However, the diagnosis of prosthetic joint SA, particularly in the hip, was sometimes longer, as seen in previous findings (*4*,*9*,*26*).

Clinical signs and conventional laboratory markers, such as elevated white blood cell count, and CRP cannot differentiate infectious from noninfectious inflammation, and these tests should not be used alone to diagnose SA. Serum PCT levels also increase in various forms of inflammation and microbial infections. PCT is <0.5 ng/mL in healthy patients but rapidly increases with systemic bacterial infections, such as SA (*27*). Some studies have reported falsely low PCT during the early phase of infection or in localized infections, such as SA (*27*). In our study, all patients had elevated CRP and 75% had elevated PCT. Synovial fluid almost always had an inflammatory appearance and often was purulent, as also described in prior studies (*4*,*6*–*10*,*28*). Positive gram staining results were reported in 80% of cases, and positive culture was reported in 93% of cases, similar to the rates from previous studies (*4*,*6*–*10*).

In the literature, pneumococcal bacteremia was complicated by joint infection in 0.3%–0.6% of cases (*10*,*29*,*30*); bacteremia was observed in 55%–100% of adults with pneumococcal SA (*4*,*6*,*8*–*10*,*16*) and appeared to be more frequent when a prosthetic joint was infected (*6*). In our study, we observed bacteremia in 69% of cases of native SA and in 80% of cases of prosthetic joint infection. The frequency of documented concurrent bacteremia emphasizes the importance of obtaining blood cultures in addition to joint fluid cultures before initiating antimicrobial therapy. Isolation of pathogenic microorganisms from both synovial fluid and blood culture can be considered the gold standard for SA diagnosis.

Most reported pneumococcal SA strains for which antimicrobial susceptibility data were available were susceptible to penicillin (*6*–*10*,*28*). A few strains with low- or high-level penicillin and ceftriaxone resistance have been reported in the literature (*6*,*7*,*10*); in our study, 25% of the strains had low-level penicillin resistance, and 12.5% had low- or high-level ceftriaxone resistance. Regardless, the frequency of low-level β-lactam resistance decreased from 2005–2010 to 2011–2016 (p<0.01). Despite the poor immunization coverage in this population, the decreased rate of resistance is related to a reduction in resistant serotypes, achieved by herd immunity.

Radiography, computed tomography, scintigraphy, and magnetic resonance imaging can be useful to assess the presence and extent of bone and joint inflammation and destruction but cannot distinguish between infections and other causes of acute inflammatory arthritis (*3*). Septic inflammation of a joint also can lead to radionuclide uptake in scintigraphy (*31*). However, diagnosis of pyogenic sacroiliitis often was made on the basis of patient history, physical examination, and positive skeletal scintigraphy or computed tomography of the sacroiliac joint.

No consensus has been reached concerning the optimal duration of intravenous antimicrobial therapy and the role of switching to oral therapy (*3*,*28*). The median duration reported in the literature ranged from 17–30.1 days for intravenous therapy and 30.6–49 days for oral antimicrobial agents (*4*,*6*,*8*–*10*). The median duration of intravenous therapy in our study was shorter, 1–23 days with a mean of 5.5 days, but the overall duration of antimicrobial therapy was comparable to reports in the literature. In several studies, penicillin was the most commonly used antimicrobial drug, then third-generation cephalosporins; gentamicin rarely was used (*7*,*8*,*10*,*28*). In contrast, 68.75% of cases in our study received a combination therapy of a β-lactam antimicrobial drug and gentamicin.

Antimicrobial therapy in the absence of drainage can be successful in certain patients. However, arthrotomy in combination with antimicrobial therapy typically is considered the best treatment for SA (*4*,*6*–*10*). In our cohort, arthrotomy was performed in 46% of cases. In the literature, when information regarding management was available, joint drainage always was performed on patients with SA in a prosthetic joint. Several cases of pneumococcal prosthetic joint SA required extended courses of antimicrobial drugs or even lifetime antimicrobial therapy, open surgical drainage, and sometimes replacement of the prosthetic joint (*3*,*7*,*8*,*26*,*28*).

Pneumococcal SA usually has a favorable prognosis when appropriate treatment is instituted rapidly (*3*,*28*). In our study and others we reviewed, most patients recovered and achieved their initial joint range of motion or had only minor sequelae with mildly reduced range of motion (*7*,*9*,*10*). Nevertheless, extensive physical damage sometimes was described (*4*), and mortality rates of up to 32% were reported in patients with pneumococcal SA (*6*–*8*,*10*,*16*). Risk of death appeared to be higher in cases of pneumococcal SA associated with bacteremia (24%) than those without bacteremia (6%) (*15*). In our study, 1 (7%) patient died with SA infection caused by a strain with low-level penicillin resistance.

Because the prevalence of antimicrobial drug–resistant *S. pneumoniae* has increased, pneumococcal vaccination has become a greater focus for public health (*12*,*15*,*17*–*21*). In the Picardie region, and in France as a whole, both PCV13 and pneumococcal 23-valent polysaccharide vaccine (PPV23) are indicated in adults with underlying conditions that predispose them to pneumococcal disease (*15*,*17*). In the Picardie Regional Pneumococcal Network, 82% (9/14) of patients with known vaccination status had not been vaccinated; of those, 5 (55.5%) had underlying conditions that would have justified pneumococcal vaccination. Of the strains isolated, 60% are covered by either PCV13 or PPV23. Of the 40% not covered by either vaccine, 5 were 23B serotype and 1 was the 24F serotype. Although several studies have demonstrated the effectiveness of pneumococcal vaccines in preventing IPD in adults (*17*,*18*,*20*,*21*), a large retrospective study of patients with pneumococcal SA reported *S. pneumoniae* serotypes 22F and 12F, both of which are covered by PPV23, frequently occur (*16*), findings that emphasize the importance of following the immunization guidelines.

In June 2010, health authorities in France introduced PCV13 for children <2 years of age; older children with >1 underlying conditions, such as innate or acquired immunodeficiency, malignancy, impaired splenic function, cochlear implants, cerebrospinal fluid leakage, or recurrent IPD; and adults at risk for pneumococcal infections, such as those with immunosuppression or history of splenectomy. According to studies in France, after the introduction of PCV13, the incidence of all IPD types decreased during 2008–2012 by 20% in adults 16–64 years of age and by 15% in patients >65 years of age (*17*). Decreases in *S. pneumoniae* isolates with reduced antimicrobial sensitivity also were noted (*15*), but the frequency of serotypes not covered by the vaccine increased (*15*,*17*). 

In our study, serotype 23B in pneumococcal SA or IPD emerged 2 years after the introduction of PCV13 (data not shown), and these strains were always penicillin susceptible. The 2 patients in our study who had *S. pneumoniae* vaccination after splenectomy were not protected against infection by serotypes 23B and 24F. Our data are consistent with trends observed in other countries (*32*–*35*). For example, Germany and the United States report a rise in serotype 23B after implementation of PCV13 (*32*–*35*) and a high proportion of the 23B isolates displayed a low-level penicillin resistance. However, the serotype 23B strain we saw in the Picardie region was always penicillin-susceptible. If confirmed by future studies, 23B and 24F serotypes should be considered when developing next-generation PCVs (*36*).

In conclusion, the prevalence of pneumococcal SA in adults in the Picardie Regional Pneumococcal Network of France increased over the 5 years reported, apparently in relation to emergence of serotype 23B. Vaccination in the region might not comply fully with the current guidelines; 60% of the strains isolated from patients in this study are covered by PCV13 and PPV23, suggesting that these pneumococcal infections could have been prevented.
